# 25(OH)D_3_-enriched or fortified foods are more efficient at tackling inadequate vitamin D status than vitamin D_3_

**DOI:** 10.1017/S0029665117004062

**Published:** 2017-11-27

**Authors:** Jing Guo, Julie A. Lovegrove, D. Ian Givens

**Affiliations:** 1 Institute for Food, Nutrition and Health, University of Reading, Reading RG6 6AR, UK; 2 Hugh Sinclair Unit of Human Nutrition, Department of Food & Nutritional Sciences, University of Reading, Reading RG6 6AP, UK

**Keywords:** Enrichment, Fortification, 25(OH)D_3_, Vitamin D_3_, Vitamin D deficiency

## Abstract

The ability to synthesise sufficient vitamin D through sunlight in human subjects can be limited. Thus, diet has become an important contributor to vitamin D intake and status; however, there are only a few foods (e.g. egg yolk, oily fish) naturally rich in vitamin D. Therefore, vitamin D-enriched foods via supplementing the animals’ diet with vitamin D or vitamin D fortification of foods have been proposed as strategies to increase vitamin D intake. Evidence that cholecalciferol (vitamin D_3_) and calcifediol (25(OH)D_3_) content of eggs, fish and milk increased in response to vitamin D_3_ supplementation of hens, fish or cows’ diets was identified when vitamin D-enrichment studies were reviewed. However, evidence from supplementation studies with hens showed only dietary 25(OH)D_3_, not vitamin D_3_ supplementation, resulted in a pronounced increase of 25(OH)D_3_ in the eggs. Furthermore, evidence from randomised controlled trials indicated that a 25(OH)D_3_ oral supplement could be absorbed faster and more efficiently raise serum 25(OH)D concentration compared with vitamin D_3_ supplementation. Moreover, evidence showed the relative effectiveness of increasing vitamin D status using 25(OH)D_3_ varied between 3·13 and 7·14 times that of vitamin D_3_, probably due to the different characteristics of the investigated subjects or study design. Therefore, vitamin D-enrichment or fortified foods using 25(OH)D_3_ would appear to have advantages over vitamin D_3_. Further well-controlled studies are needed to assess the effects of 25(OH)D_3_ enriched or fortified foods in the general population and clinical patients.

Vitamin D is usually synthesised in skin that is exposed to UV radiation, which has led to the term ‘sunshine vitamin’^(^^1^^)^. Traditionally, the primary role of vitamin D is related to calcium absorption and bone health. Children and adults with vitamin D deficiency have an increased risk of developing rickets or osteomalacia^(^^2^^)^. Recently, a resurgence of childhood rickets has highlighted the need for adequate vitamin D status in many parts of the world^(^^3^^–^^5^^)^. Furthermore, mounting evidence from epidemiological studies indicates that vitamin D status is inversely associated with the risk of CVD, cancers and diabetes^(^^1^^,^^6^^)^, although there is some uncertainty about what defines an adequate vitamin D status^(^^7^^)^.

Vitamin D deficiency is prevalent and is considered a serious issue throughout the world^(^^8^^–^^10^^)^, even in sunnier climates such as Australia and New Zealand^(^^11^^)^. Recently, the Scientific Advisory Committee on Nutrition^(^^7^^)^ reported that in the UK, 22–24 % of adults aged 19–64 years, and 17–24 % of those ≥65 years were vitamin D deficient (plasma 25-hydroxyvitamin D_3_ (25(OH)D_3_) <25 nmol/l). There are several factors that have contributed to the low vitamin D status commonly seen today, such as lifestyle changes (increased indoor lifestyle, sun screens use) and human characteristics (e.g. ageing, clothing, increased obesity, low-fat diet trend)^(^^12^^)^. Therefore, foods that contribute to vitamin D intake have become more important than before. However, there are only a few foods naturally rich in vitamin D, such as oily fish and egg yolks^(^^13^^)^.

The aim of this review is first to critically evaluate the existing evidence on whether the vitamin D content of animal-derived foods can be increased by feeding cholecalciferol (vitamin D_3_) and/or calcifediol (25(OH)D_3_) supplements to laying hens, fish and cows. Second, the present review summaries evidence from the human randomised controlled trials (RCT), which include the effects of 25(OH)D_3_ supplementation on increasing serum/plasma 25(OH)D_3_ concentration.

## Vitamin D absorption, synthesis and metabolism

Generally, the term vitamin D refers to both vitamin D_2_ and vitamin D_3_. Vitamin D_2_ is produced by fungi, while vitamin D_3_ is produced by human subjects and animals^(^^14^^)^. Human subjects usually synthesise vitamin D_3_ in the skin^(^^15^^)^ where 7-dehydrocholesterol in the epidermis is converted to pre-vitamin D_3_ when skin is exposed to sunlight. Then, pre-vitamin D_3_ undergoes a temperature-dependent isomerisation to vitamin D_3_ over a period of approximately 3 d^(^^6^^)^. Vitamin D (vitamin D_2_ or vitamin D_3_) can also be obtained from the diet^(^^15^^)^ and it is absorbed with long-chain TAG in the small intestine^(^^16^^)^. It is then incorporated into chylomicrons and transported in lymph to the blood and into the general circulation^(^^17^^)^.

After entering the circulation, there are two hydroxylation reactions to convert vitamin D to the biologically active form^(^^6^^)^. The first hydroxylation reaction is in the liver where vitamin D is hydroxylated to 25(OH)D by the vitamin D-25-hydroxylase enzyme. The second hydroxylation reaction is in the kidney where 25(OH)D is converted to 1,25(OH)_2_D by 25-hydroxyvitamin D-1*α*-hydroxylase^(^^6^^)^, and the 1,25(OH)_2_D metabolite is the biologically active form of vitamin D^(^^18^^)^.

## Foods of animal origin as dietary sources of vitamin D

Vitamin D content of vitamin D-enriched foods can differ considerably between food retailers. One US retail study analysed the vitamin D content of egg yolks collected from twelve individual retail supermarkets across the country and reported a broad range of vitamin D_3_ and 25(OH)D_3_ concentrations of 9·7–18 and 4·3–13·2 µg/kg, respectively^(^^19^^)^. In addition, our recent UK retail study^(^^20^^)^ showed vitamin D_3_ and 25(OH)D_3_ concentrations of eggs were significantly different depending on the egg production systems. Egg yolks produced by birds kept in indoor systems had much lower concentrations (40·2 (se 3·1) µg/kg) of vitamin D_3_ than the egg yolks produced from outdoor systems (57·2 (se 3·2) µg/kg), while 25(OH)D_3_ concentrations of the eggs were higher in organic eggs only. Similarly, the vitamin D contents of fish have been shown to vary according to the production systems. The study of Lu *et al.*^(^^21^^)^ indicated the vitamin D_3_ content of wild salmon to be three times higher than that of farmed salmon; however, the 25(OH)D_3_ content of the salmon was not measured. In addition, other studies^(^^22^^,^^23^^)^ have shown the 25(OH)D_3_ content of several species of marine and freshwater fish to be <0·02 µg/100 g. Therefore, foods generally regarded as rich sources of vitamin D may not be sustainable vitamin D contributors for the general population, due to variability in vitamin D content, which in turn may be influenced by production systems or different species (genotype). Furthermore, the National Diet and Nutrition Survey of the UK^(^^24^^)^ reported that the average daily intake of vitamin D for adults was 3·1 µg for men and 2·6 µg for women, which is much lower than the UK vitamin D reference nutrition intake of 10 µg/d^(^^7^^)^. Therefore, vitamin D-enriched or fortified foods are needed to ensure an adequate vitamin D intake for the general population.

## Enrichment of animal-derived foods as dietary sources of vitamin D

### Vitamin D-enriched eggs

In general, there are two main methods to enrich the vitamin D content of eggs: increased sunlight exposure and vitamin D supplementation of the birds’ diet. Because hens can synthesise vitamin D from natural sunlight exposure, free-range egg production system may be an inexpensive way to increase their vitamin D content. A study by Kuhn *et al.* assigned laying hens to a free-range treatment or an indoor treatment for over 4 weeks and found that eggs from the free-range group, which were exposed to sunlight, had significantly higher vitamin D_3_ content (mean 14·3 µg/100 g DM) than eggs from the indoor group (mean 3·8 µg/100 g DM)^(^^25^^)^. Furthermore, there are several studies which have shown that the vitamin D_3_ content of eggs can be enhanced by feeding vitamin D_3_ supplements to the hens (Table 1)^(^^26^^–^^32^^)^. The results of all studies revealed that egg yolk vitamin D_3_ concentration was efficiently increased by vitamin D_3_ dietary supplementation. The study of Yao *et al.* showed a linear dose–response relationship existed between vitamin D_3_ dietary supplementation and vitamin D_3_ concentrations of egg yolks^(^^30^^)^. Moreover, as 25(OH)D_3_ is a metabolite of vitamin D_3_, the 25(OH)D_3_ content in eggs can also be enhanced by supplementing the birds’ diet with vitamin D_3._ However, the response in 25(OH)D_3_ content of egg yolk is much less than that of vitamin D_3._ Browning and Cowieson^(^^31^^)^ showed that a 4-fold increase in vitamin D_3_, and a 2-fold increase in 25(OH)D_3_ in egg yolk resulted from a 4-fold increase in the vitamin D_3_ in the diet (62·5–250 µg/kg). Similarly, evidence from another study showed that the vitamin D_3_ in egg yolk was increased approximately 7-fold as a result of feeding a diet with a 3·5-fold higher vitamin D_3_ content (from 62·4 to 216 µg/kg), while the corresponding increase in 25(OH)D_3_ content was only about 1·5-fold^(^^26^^)^.

**Table 1.** Summary of enrichment studies investigating the impact of adding vitamin D to the diet of laying hens on the vitamin D content of egg yolks

References	Vitamin D supplement (μg/kg)		Feeding duration (weeks)	Vitamin D concentration of egg yolk (μg/100 g)	
	Vitamin D_3_	25(OH)D_3_		Vitamin D_3_	25(OH)D_3_
Mattila *et al.*^(^^26^^)^	26·6	–	6	1·4	0·5
	62·4	–	6	3·5	0·9
	216·0	–	6	22·0	1·5
Mattila *et al.*^(^^27^^)^	280·0	–	4	30·0	1·9
Mattila *et al.*^(^^28^^)^	62·5	–	4	3·8	–
	150·0	–	4	13·6	–
	375·0	–	4	33·7	–
Browning and Cowieson^(^^31^^)^	62·5	–	9	6·5	1·6
	125·0	–	9	10·5	2·1
	250·0	–	9	26·2	3·0
Yao *et al.*^(^^30^^)^	55·0	–	3	3·0	–
	242·5	–	3	21·6	–
	430·0	–	3	41·0	–
	617·5	–	3	60·3	–
	2555·0	–	3	870·4	–
Browning and Cowieson^(^^31^^)^	62·5	0	9	6·5	1·6
	62·5	34·5	9	6·0	3·3
	62·5	69·0	9	4·9	4·5
	125·0	0	9	10·5	2·1
	125·0	34·5	9	7·4	4·5
	125·0	69·0	9	8·1	5·8
	250·0	0	9	26·2	3·0
	250·0	34·5	9	23·6	3·7
	250·0	69·0	9	30·9	8·1
Mattila *et al.*^(^^29^^)^	–	55·0	6	≤0·2	2·1
	–	122·0	6	≤0·2	4·3
Duffy *et al.*^(^^32^^)^	37·5	–	4	1·0*	1·9*
	75·0	–	4	2·0*	1·9*
	37·5	37·5	4	1·3*	3·6*
		75·0	4	0·7*	4·4*

25(OH)D_3_, 25-hydroxyvitamin D_3_.

* Vitamin D content per egg.

There are only a few studies^(^^29^^,^^31^^,^^32^^)^ examining the effect of feeding birds with diets supplemented with 25(OH)D_3._ In the EU, 25(OH)D_3_ has only recently been authorised for addition to poultry diets, and the maximum content of the vitamin D_3_ and 25(OH)D_3_ combination for laying hens is 80 µg/kg^(^^33^^,^^34^^)^. It is of note that most of vitamin D supplementation studies^(^^27^^–^^31^^)^, summarised in Table 1, had higher vitamin D doses than the EU diet limit^(^^33^^)^, thus, the potential for increasing vitamin D in eggs by adding vitamin D to the diet of laying hens is limited by EU regulations. Browning and Cowieson^(^^31^^)^ and Duffy *et al.*^(^^32^^)^ both showed an addition of 25(OH)D_3_ to the vitamin D_3_ supplement resulted in the elevation of the 25(OH)D_3_ content of the egg yolk, but there was no significant increase in the vitamin D_3_ content of the egg yolk. Other studies investigated dietary supplementation with 25(OH)D_3_^(^^29^^,^^32^^)^, and showed that only egg yolk 25(OH)D_3_ was increased, but not vitamin D_3_. Therefore, we speculate that 25(OH)D_3_ in the diet can be absorbed directly by laying hens without transfer to vitamin D_3_ in the circulation.

### Vitamin D-enriched fish

There are very few studies on enriching the vitamin D content of fish (Table 2)^(^^35^^–^^38^^)^. Mattila *et al.* fed rainbow trout with different doses of vitamin D_3_ supplements up to 539 µg/kg, but no significant differences in the vitamin D_3_ content of the fish fillet were observed^(^^37^^)^. In contrast, the study of Horvli *et al.* with Atlantic salmon showed a dose–response relationship between the vitamin D_3_ in the diet up to 28·68 mg/kg and vitamin D_3_ in the fish meat^(^^35^^)^. Similar high vitamin D_3_ supplementation doses were reported in another two studies^(^^36^^,^^38^^)^, which also showed that elevated vitamin D_3_ content of the fish liver or whole fish had been achieved by supplemental vitamin D_3_ in the diet. However, 25(OH)D_3_ contents of the enriched fish were not measured in these studies^(^^35^^–^^38^^)^, and the lack of evidence on the effects by feeding fish with 25(OH)D_3_ on the vitamin D content of the fish warrants further research. Again, supplement doses of the listed studies^(^^35^^–^^38^^)^ in Table 2 were over the EU diet limit for farmed fish of 75 µg/kg^(^^33^^)^, which will limit application in the market.

**Table 2.** Summary of enrichment studies investigating the impact of vitamin D supplemental fish feeding on vitamin D content of fish

References	Vitamin D_3_ supplement (μg/kg)	Feeding duration (weeks)	Vitamin D_3_ of fish (μg/100 g)
Horvli *et al.*^(^^35^^)^	40	11	1 (fillet)
	2210	11	21 (fillet)
	28 680	11	210 (fillet)
Vielma *et al.*^(^^36^^)^	62·5	12	1 (liver)
	6250	12	73 (liver)
	62 500	12	6900 (liver)
Mattila *et al.*^(^^37^^)^	89	16	6–15 (fish fillet)
	174	16	6–10 (fish fillet)
	539	16	7–16 (fish fillet)
Graff *et al.*^(^^38^^)^	200	9	≤25 (whole fish)*
	5000	9	80 (whole fish)*
	57 000	9	650 (whole fish)*

* Estimated from graph.

### Vitamin D-enriched milk

A few studies have investigated the longer term effect of supplemental vitamin D_3_ on the vitamin D content of the milk; the summary of these studies is presented in Table 3^(^^39^^–^^42^^)^. Hollis *et al.* showed a 10-fold enhancement of vitamin D_3_ intake from 100 to 1000 µg/d resulted in a 7·5-fold increased vitamin D_3_ concentration of the milk and a 2-fold increase in 25(OH)D_3_^(^^39^^)^. Moreover, McDermott *et al.* compared three different doses of vitamin D_3_ with a control diet, and showed an increased concentration of vitamin D_3_ and 25(OH)D_3_ in the milk^(^^41^^)^. However, the relationship between increasing dietary vitamin D_3_ doses and milk vitamin D_3_ or 25(OH)D_3_ concentrations were not linear. Furthermore, the study of Weiss *et al.* investigated the effect of feeding 450 µg/d vitamin D_3_ to pre-calving cows for 13 d which resulted in concentrations of vitamin D_3_ and 25(OH)D_3_ in the milk ranging from 0·33–0·45 to 0·36–1·02 µg/l, respectively^(^^42^^)^. In addition, the study included a diet treatment of 6 mg vitamin D_3_ with a cation–anion difference of −138 mEq/kg daily for 13 d; the concentrations of 25(OH)D_3_ in the milk were increased but the treatment effect disappeared after 28 d. Therefore, evidence from the limited number of studies^(^^39^^–^^42^^)^ demonstrated that milk vitamin D concentrations can be increased by feeding dairy cows with vitamin D supplements. However, it is of note that the highest milk vitamin D_3_ and 25(OH)D_3_ concentrations were 0·47 and 3·69 µg/l, respectively (Table 3), which for one typical milk serving of 200 ml only contributes 0·09 and 0·74 µg vitamin D_3_ and 25(OH)D_3_, respectively, well below the current UK vitamin D reference nutrition intake of 10 µg/d^(^^7^^)^. Furthermore, the doses of vitamin D in those studies^(^^41^^,^^42^^)^ were much higher than the maximum allowed vitamin D content in EU (0·01 mg/kg diet at 880 g DM/kg approximately equivalent to 2·27 mg/d)^(^^34^^)^, which imposes an even greater restriction on the possibility of increasing vitamin D in milk by adding vitamin D supplements in the diet of dairy cows.

**Table 3.** Summary of enrichment studies investigating the impact of vitamin D supplementation to the diet of dairy cows on vitamin D content of milk

References	Supplements to diet (μg/d)			Vitamin D concentration of milk (μg/l)		
	Vitamin D_3_	25(OH)D_3_	Feeding duration	Vitamin D_3_	25(OH)D_3_	1,25(OH)_2_ D_3_
Hollis *et al.*^(^^39^^)^	100	–	NA	0·04	0·37	0·01
	1000	–	NA	0·32	0·68	0·004
Reeve *et al.*^(^^40^^)^	375	–	30 d	0·28	0·15	0·01
Mcdermott *et al.*^(^^41^^)^	0	–	14 weeks	0·08	0·25	0·10
	250	–	14 weeks	0·20	0·43	0·03
	1250	–	14 weeks	0·15	0·75	0·13
	6250	–	14 weeks	0·33	0·93	0·10
Weiss *et al.*^(^^42^^)^	450	–	13 d before calving	0·33–0·47	0·36–1·02	–
	–	DCAD + 6000	13 d before calving	–	0·61–3·69	–

25(OH)D_3_, 25-hydroxyvitamin D_3_; 1,25(OH)_2_D_3_, 1,25 dihydroxyvitamin D_3_; DCAD, dietary cation–anion difference of −138 mEq/kg.

## Evidence from human dietary intervention studies with vitamin D-enriched animal-derived foods

Despite numerous animal-based vitamin D-enrichment studies on vitamin D in eggs, fish and milk, there are few RCT on the effect of consuming vitamin D-enriched foods on the vitamin D status of the consumer. To our knowledge, only one recent study has investigated the weekly effect of consuming seven vitamin D_3_ or seven 25(OH)D_3_-enriched eggs on vitamin D status compared with commercial eggs of ≤2 egg/week^(^^43^^)^. After 8 weeks follow-up in winter, the results showed that while the serum 25(OH)D of the subjects who consumed commercial eggs decreased from a baseline of 41 (sd 14·1) nmol/l to 35 (sd 11·4) nmol/l, the serum 25(OH)D of subjects who consumed vitamin D_3_-enriched eggs or 25(OH)D_3_-enriched eggs was maintained. The serum 25(OH) D concentrations of subjects who consumed vitamin D_3_- or 25(OH)D_3_-enriched eggs were 50 (sd 21·4) nmol/l and 49 (sd 16·5) nmol/l, respectively. However, there was no significant difference between vitamin D_3_- and 25(OH)D_3_-enriched egg consumption on serum 25(OH)D concentrations.

Although there are a limited number of human dietary intervention studies on vitamin D-enriched foods, the study of Mattila *et al.*^(^^29^^)^ demonstrated that the effect of foods enriched with either vitamin D_3_ or 25(OH)D_3_ on human vitamin D status depended on their relative effectiveness of raising serum or plasma 25(OH)D concentrations. A previous study^(^^44^^)^ indicated that there was no consensus on the relative effectiveness of 25(OH)D_3_ compared with vitamin D_3_ for raising human serum or plasma 25(OH)D_3_ concentrations. Furthermore, UK food composition tables^(^^45^^)^ indicate that there is no certainty on the relative potency of 25(OH)D_3_ compared with vitamin D_3_, although it was assumed that 25(OH)D_3_ had a potency of five times that of vitamin D_3_ for calculating the total vitamin D of foods^(^^45^^)^.

## Human intervention studies on the relative effects of calcifediol and cholecalciferol supplementation on vitamin D status

### Heterogeneity of intervention studies

Eleven RCT that investigated the effects of 25(OH)D_3_ relative to vitamin D_3_ were identified^(^^46^^–^^56^^)^ (Table 4). Nine studies administered 25(OH)D_3_ supplementation only, except two studies which provided a combination supplement of 25(OH)D_3_ and calcium^(^^46^^,^^49^^)^. Five of the eleven studies^(^^47^^,^^49^^–^^52^^)^ supplemented 25(OH)D_3_ to generally healthy subjects, whereas the other six studies^(^^46^^,^^48^^,^^53^^–^^56^^)^ supplemented 25(OH)D_3_ to clinical patients. Most studies reported the serum or plasma 25(OH)D concentration at both the beginning and end of the treatment, except one study^(^^55^^)^, which only reported the 25(OH)D concentration at the end of the treatment. In terms of the vitamin D status measurement, most studies measured total 25(OH)D concentration, except two studies^(^^49^^,^^52^^)^, which measured 25(OH)D_3_. For the characteristics of the investigated subjects, five studies included both men and women^(^^46^^,^^48^^,^^51^^,^^53^^,^^55^^)^, while the other studies only included men or women. In addition, most studies reported the age and BMI of the subjects, except two studies^(^^46^^,^^48^^)^ that did not report the BMI range.

**Table 4.** Summary of study details and serum 25, hydroxyvitamin D (25(OH)D) concentration in long-term randomised controlled trials with calcifediol (25 hydroxyvitamin D_3_ (25(OH)D_3_)) supplementation in adults (order by year)

References	Subjects characteristics (trail time during the year, subjects (sex), age, BMI)	25(OH)D_3_ supplementation group					Control group (if available)				
		Duration	25(OH)D_3_ treatment	*n*	Baseline 25(OH)D (nmol/l)	Endpoint 25(OH)D (nmol/l)	Duration	Vitamin D_3_ treatment	*n*	Baseline 25(OH)D (nmol/l)	Endpoint 25(OH)D (nmol/l)
Hahn *et al.*^(^^46^^)^	Whole year, patients (women and men) with glucocorticoid-induced osteopenia 46 years, BMI (NA*)	18 months	40 µg/d + 500 mg calcium/d	9	39	205					
Barger-Lux *et al.*^(^^47^^)^	January–April, men 28 years, 26 kg/m^2^	4 weeks	10 µg/d	7	67	107	8 weeks	25 µg/d	13	67	96
		4 weeks	20 µg/d	6	67	143	8 weeks	250 µg/d	10	67	213
		4 weeks	50 µg/d	4	67	273	8 weeks	1250 µg/d	14	67	710
Jean *et al.*^(^^48^^)^	March–September, haemodialysis patients (women and men) 67 years, BMI (NA)	6 months	16 µg /d	149	30	126					
Cavalli *et al.*^§^^(^^49^^)^	April–July, postmenopausal women 65–75 years, 25 kg/m^2^	12 weeks	125 µg/week + 500 mg calcium/d	25	50	76					
		12 weeks	250 µg/month + 500 mg calcium/d	28	51	70					
		12 weeks	500 µg/month + 500 mg calcium/d	27	52	77					
Russo *et al.*^(^^50^^)^	January–April, women (7 premenopausal and 11 postmenopausal), 24–72 years, 24 kg/m^2^	16 weeks	500 µg/month	18	45	105^†^					
Cashman *et al.*^(^^51^^)^	January–April, women and men, 57 years, 29 kg/m^2^	10 weeks	20 µg/d	12	38	135	10 weeks	20 µg/d	13	50	69
Jetter *et al.*^‡^^§^^(^^52^^)^	January–July, postmenopausal women 50–70 years, 18–29 kg/m^2^	16 weeks	20 µg/d	5	31	173	16 weeks	20 µg/d	5	35	77
Catalano *et al.*^(^^54^^)^	September–March, osteopenic and dyslipidaemic postmenopausal women 59 years, 27 kg/m^2^	24 weeks	140 µg once weekly	29	56	126	24 weeks	140 µg once weekly	28	51	61
Banon *et al.*^(^^53^^)^	Whole year, patients (women and men) had HIV-infected, 44 years, 15–44 kg/m^2^	Summer	400 µg once/month	123	37	86	Summer	NA	242	53	99
		Fall	400 µg once/month	123	37	69	Fall	NA	242	53	84
		Winter	400 µg once/month	123	37	45	Winter	NA	242	53	55
		Spring	400 µg once/month	123	37	57	Spring	NA	242	53	78
Ortego-Jurado *et al.*^(^^55^^)^	Whole year, patients (women and men) had autoimmune diseases, undergoing glucocorticoids therapy, 56 years, 28 kg/m^2^	Spring–summer	8·85 µg/d	49	NA	84	Spring–summer	20 µg/d	86	NA	71
		Fall–winter	8·85 µg/d	49	NA	89	Fall–winter	20 µg/d	86	NA	61
Navarro-Valverde *et al.*^(^^56^^)^	Whole year, postmenopausal osteoporotic women, 67 years, 26 kg/m^2^	6 months	20 µg/d	10	37	161	6 months	20 µg/d	10	41	80
		12 months	20 µg/d	10	37	188	12 months	20 µg/d	10	41	86
		6 months	266 µg once/week	10	38	214					
		12 months	266 µg once/week	10	38	233					
		6 months	266 µg once/2 weeks	10	40	165					
		12 months	266 µg once/2 weeks	10	40	211					

* NA, not available.

† Estimated from graph.

‡ Same study of (Jetter *et al.*^(^^52^^)^) and (Bischoff-Ferrari *et al.*^(^^62^^)^).

§ Study has measured vitamin D status as 25(OH)D_3_.

### Acute pharmacokinetic action of cholecalciferol and calcifediol

An early study provided meals with single doses of 25(OH)D_3_ of 1·5, 5 or 10 µg/kg body weight to generally healthy subjects and showed that the peak serum 25(OH)D_3_ concentration was reached within 4–8 h after ingestion^(^^57^^)^. A later study by Jetter *et al.* compared the pharmacokinetic absorption of vitamin D_3_ and 25(OH)D_3_ by providing a single dose of 20 µg vitamin D_3_ or 20 µg 25(OH)D_3_ to postmenopausal women^(^^52^^)^. The time to reach maximum plasma 25(OH)D_3_ concentration was 22 and 11 h for vitamin D_3_ and 25(OH)D_3_, respectively. In addition, the peak concentration of plasma 25(OH)D_3_ (44 nmol/l) from 25(OH)D_3_ supplementation was higher than vitamin D_3_ supplementation (35 nmol/l), although they were not significantly different. This study further compared the effect of a higher single dose of 140 µg vitamin D_3_ and 140 µg 25(OH)D_3_ with the time to reach peak plasma 25(OH)D_3_ being 21 and 4·8 h for vitamin D_3_ and 25(OH)D_3_ supplementation, respectively^(^^52^^)^. In addition, the maximum plasma concentration of 25(OH)D_3_ for 25(OH)D_3_ treatment (100 nmol/l) was significantly higher than for vitamin D_3_ treatment (44 nmol/l). These results suggest that 25(OH)D_3_ was absorbed more quickly than vitamin D_3_ possibly because 25(OH)D_3_ has higher solubility in aqueous media than vitamin D_3_ due to its more polar chemical structure^(^^58^^)^. Furthermore, as this metabolite of vitamin D_3_ is produced in the liver, the hepatic metabolism of vitamin D_3_ to 25(OH)D_3_ is circumvented and consequently the conversion from vitamin D_3_ to 25(OH)D_3_ would be negligible^(^^59^^)^. In patients with liver disease who had an impaired ability to synthesise 25(OH)D_3_ from vitamin D_3_^(^^60^^)^, the study of Sitrin and Bengoa^(^^61^^)^ verified that 25(OH)D_3_ could be absorbed more efficiently than vitamin D_3_ after oral supplementation. Therefore, supplementation with 25(OH)D_3_ is not only more efficient at increasing vitamin D status in generally healthy people, but may also have a specific role in tackling lower vitamin D status in patients who are suffering from liver diseases.

### Chronic effects and relative effectiveness of cholecalciferol and calcifediol treatments

Regarding the expected higher biological effect of 25(OH)D_3_ in raising serum or plasma 25(OH)D level after long-term administration, several studies have confirmed that oral consumption of 25(OH)D_3_ is highly effective in raising serum or plasma 25(OH)D level (Table 4)^(^^46^^–^^56^^)^. However, the majority of the evidence in support of a higher impact of 25(OH)D_3_ supplementation compared with vitamin D_3_ on serum or plasma 25(OH)D_3_ level is from only four studies^(^^51^^,^^52^^,^^54^^,^^56^^)^ where both 25(OH)D_3_ and vitamin D_3_ treatments were included in the same study (Table 5). The study of Barger-Lux *et al.*^(^^47^^)^ provided three different doses of vitamin D_3_ (25, 250, 1250 µg/d) or 25(OH)D_3_ (10, 20, 50 µg/d) to the participants for 8 and 4 weeks, respectively. However, the effects of 25(OH)D_3_ and vitamin D_3_ treatments were not directly comparable as the interventions were not at the same dose or treatment time. Thus, the study of Barger-Lux *et al.*^(^^47^^)^ was excluded from the relative effectiveness analysis. In order to compare the relative effectiveness of 25(OH)D_3_ and vitamin D_3_ supplementation on raising serum or plasma 25(OH)D concentrations, a dose–response factor was calculated for each μg of orally consumed 25(OH)D_3_ or vitamin D_3_ in four studies^(^^51^^,^^52^^,^^54^^,^^56^^)^. The dose–response factors of 25(OH)D_3_ and vitamin D_3_ were calculated by using endpoint 25(OH)D concentration minus baseline 25(OH)D concentration, divided by the dose of the supplementation (dose–response factor = Δ serum/plasma (mmol/l)/dose (μg)). Then, the relative effectiveness of 25(OH)D_3_ to vitamin D_3_ was calculated by dividing the dose–response factor of 25(OH)D_3_ by that of vitamin D_3_.

**Table 5.** Summary of randomised controlled trials with both calcifediol (25 hydroxyvitamin D_3_ (25(OH)D_3_)) and vitamin D_3_ in adults to calculate the relative effectiveness of 25(OH)D_3_ and vitamin D_3_ supplementation in raising serum 25, hydroxyvitamin D (25(OH)D) level

References	Treatment (dose, duration)	Serum 25(OH)D raising (nmol/l) per 1 μg*	Relative effectiveness^†^
Cashman *et al.*^(^^51^^)^	20 µg 25(OH)D_3_/d × 10 weeks	4·82^a^	4·99
	20 µg vitamin D_3_/d × 10 weeks	0·97^b^	
Jetter *et al.*^(^^52^^)^	20 µg 25(OH)D_3_/d × 15 weeks	7·12^a^	3·40
	20 µg vitamin D_3_/d × 15 weeks	2·51^b^	
Catalano *et al.*^(^^54^^)^	140 µg 25(OH)D_3_/week × 24 weeks	0·50^a^	7·14
	140 µg vitamin D_3_/week × 24 weeks	0·07^b^	
Navarro-Valverde *et al.*^(^^56^^)^	20 µg 25(OH)D_3_/d × 6 months	6·19^a^	3·13
	20 µg vitamin D_3_/d × 6 months	1·98^b^	
	20 µg 25(OH)D_3_/d × 12 months	7·54^a^	3·29
	20 µg vitamin D_3_/d × 12 months	2·29^b^	

* Dose–response factor = Δ serum/plasma (mmol/l)/dose (μg).

† Relative effectiveness = a/b within same study.

The highest relative effectiveness was found in the study by Catalano *et al.*^(^^54^^)^. Weekly treatment of 140 µg 25(OH)D_3_ or 140 µg vitamin D_3_ supplements was provided to osteopenic and dyslipidaemic postmenopausal women for 24 weeks. Supplementation with 25(OH)D_3_ raised serum 25(OH)D from a baseline of 56–126 nmol/l, while vitamin D_3_ treatment increased serum 25(OH)D to a lower extent, from baseline 51 to 61 nmol/l. Thus, the relative effectiveness factor derived from this study was 7·14, i.e. dietary 25(OH)D_3_ was 7·14 times more effective at increasing serum 25(OH)D than dietary vitamin D_3_.

Vitamin D dietary recommendations are generally between 10 and 20 µg/d^(^^10^^)^, yet, there are few studies which have compared the effectiveness of dietary 25(OH)D_3_ and vitamin D_3_ using doses of 20 µg in their treatments. Cashman *et al.*^(^^51^^)^ provided daily supplements of 20 µg vitamin D_3_ or 20 µg 25(OH)D_3_ to adult men and women with a mean age of 57 years and with baseline serum 25(OH)D of 28·9 nmol/l during winter. After 10 weeks of supplementation, the subjects’ serum 25(OH)D increased to 135 and 69 nmol/l for the 25(OH)D_3_ and vitamin D_3_ treatments, respectively. A relative effectiveness factor of 4·99 was calculated representing the relative effectiveness of each μg of dietary 25(OH)D_3_ relative to dietary vitamin D_3_ for raising serum 25(OH)D concentration. However, lower relative effectiveness factors were achieved in other studies using the same dose of 20 µg vitamin D_3_ and 25(OH)D_3_. Jetter *et al.* supplemented healthy postmenopausal women with 20 µg 25(OH)D_3_ or 20 µg vitamin D_3_ for 16 weeks during the winter^(^^52^^)^. They found that for the 25(OH)D_3_ treatment, plasma 25(OH)D_3_ increased to 173 nmol/l from a baseline of 31 nmol/l, whereas for the vitamin D_3_ treatment, plasma 25(OH)D_3_ increased to 77 nmol/l from a baseline level of 35 nmol/l. The relative effectiveness factor of each μg of 25(OH)D_3_ was 3·40 compared with vitamin D_3_ in raising plasma 25(OH)D_3_ level. A similar low relative effectiveness factor was found in another study where post-menopausal osteoporotic women were given either 20 µg vitamin D_3_ or 20 µg 25(OH)D_3_ over 6 or 12 months^(^^56^^)^. The serum concentration of 25(OH)D for the 25(OH)D_3_ treatment reached 161 and 188 nmol/l from a baseline of 37 nmol/l after 6 or 12 months administration, respectively, while the comparable values for the vitamin D_3_ treatment were an increase to 80 and 86 nmol/l from a baseline of 41 nmol/l. So the relative effectiveness factor of 25(OH)D_3_ relative to vitamin D_3_ treatment at 6 and 12 months were 3·13 or 3·29, respectively.

In summary, of the studies reviewed, the relative effectiveness of 25(OH)D_3_ to vitamin D_3_ for raising vitamin D status (Table 5), ranged from 3·13 to 7·14. Previous studies have demonstrated that the season may have influences on vitamin D status^(^^13^^,^^14^^)^. There were two studies conducted during the winter which may have minimised any confounding influence of cutaneous vitamin D synthesis from UV radiation ^(^^47^^,^^51^^)^. Other studies have longer intervention periods of 6 months or more, which could not have avoided some cutaneous synthesis. Furthermore, baseline status may be another factor that influences the relative effectiveness factor. The study of Catalano *et al.* had the highest factor of 7·14 in the present review, and the baseline concentration of 25(OH)D of the study participants was higher (>50 nmol/l) than the others^(^^54^^)^. Therefore, the different relative effectiveness seen in different studies may be due to the different characteristics or genotypes of the subjects, or different study designs.

Overall, evidence suggests that dietary 25(OH)D_3_ can more effectively increase serum 25(OH)D concentrations than vitamin D_3_ and may also be absorbed faster reaching a serum or plasma 25(OH)D plateau earlier than vitamin D_3_ supplementation. Furthermore, supplementation with 25(OH)D_3_ may also have more benefits to human health compared with vitamin D_3_ in a general healthy population. Bischoff-Ferrari *et al.* reported that 20 µg 25(OH)D_3_ supplementation over 4 months led to a 5·7 mmHg decrease in systolic blood pressure and improvements in several markers of innate immunity in healthy postmenopausal women^(^^62^^)^.

For patients with different diseases and receiving long-term medication, studies^(^^63^^–^^65^^)^ showed that several drugs (e.g. antiepileptic agents, glucocorticoids, antiretroviral or anti-oestrogen drugs) interfered with vitamin D metabolism, which resulted in patients being more likely to have low vitamin D status. Thus, it is not only important to increase vitamin D status in the generally healthy population but also in patients with specific illnesses and receiving certain medication. Therefore, the studies using 25(OH)D_3_ treatments in patients were also summarised in Table 4^(^^46^^,^^48^^,^^53^^–^^56^^)^, and those studies consistently reported that chronic 25(OH)D_3_ supplementation effectively increased serum 25(OH)D concentrations. For example, Ortego-Jurado *et al.* showed a lower daily dose of 8·85 µg 25(OH)D_3_ to be more effective than a 20 µg dose of vitamin D_3_ for increasing vitamin D status in patients with autoimmune disease who were treated with a low dose of glucocorticoids throughout the year^(^^55^^)^. Similarly, the study of Banon *et al.* showed that a monthly dose of 400 µg 25(OH)D_3_ was safe and effective at improving vitamin D status of HIV-infected patients throughout the year^(^^53^^)^.

Furthermore, supplementation with 25(OH)D_3_ may have additional benefits on patients’ health. Previously, 25(OH)D_3_ was recommended for patients with kidney disease since 25(OH)D_3_ has a direct action on bone metabolism^(^^66^^)^. Hahn *et al.* provided a daily 40 µg 25(OH)D_3_ and 500 mg calcium supplement to patients who had glucocorticoid-induced osteopenia for 18 months^(^^46^^)^. The treatment markedly increased vitamin D status from 39 to 205 nmol/l. In addition, this study showed that the 25(OH)D_3_ treatment improved mineral and bone metabolism. Jean *et al.* also offered haemodialysis patients who suffered from vitamin D deficiency with a daily dose of 16 µg 25(OH)D_3_ for 6 months; vitamin D status reached 126 nmol/l from 30 nmol/l, at the same time 25(OH)D_3_ supplementation corrected the excess bone turnover^(^^48^^)^. Similarly, a study by Catalano *et al.*^(^^54^^)^ provided 140 µg 25(OH)D_3_ supplements for 24 weeks to osteopenic and dyslipidaemic postmenopausal women, and results showed that 25(OH)D_3_ improved plasma lipid levels (increased HDL-cholesterol (*P* = 0·02) and decreased LDL-cholesterol (*P* = 0·02)) in osteopenic and dyslipidaemic postmenopausal women when added to an ongoing atorvastatin treatment.

As an alternative to vitamin D-enriched foods, vitamin D fortification of foods may also be an option for tackling vitamin D deficiency throughout the world. In general, fortification of foods refers to mandatory and voluntary fortification. The contribution of vitamin D-fortified foods to vitamin D intake by the public varies considerably between countries as there are different food standard policies^(^^10^^)^, and in practice, vitamin D_2_ or vitamin D_3_ are used for fortification. Evidence from one previous meta-analysis of RCT showed that vitamin D_3_ supplementation is more effective at raising vitamin D status than vitamin D_2_^(^^67^^)^. However, a further comprehensive systematic review and meta-analysis of thirty-three RCT^(^^68^^)^ showed that the effect of vitamin D_3_ supplement on serum 25(OH)D_3_ response was limited by the supplemental dose, duration, age of subjects and baseline level. In addition, the meta-analysis showed a greater serum or plasma 25(OH)D increase when the intervention study used a dose of 20 µg/d vitamin D_3_ or even higher, with subjects aged >80 years and an administration period of at least 6–12 months or subjects had lower baseline 25(OH)D status (<50 nmol/l) than subjects aged <80 years, administration period <6 months or subjects had higher baseline 25(OH)D status (≥50 nmol/l)^(^^68^^)^. Therefore, better strategies are needed to raise vitamin D status of the public throughout life, and 25(OH)D_3_-fortified foods warrant further research.

## Conclusions

Vitamin D insufficiency has become a world problem, especially where sunlight exposure is limited by geographic reasons (latitude), personal characteristics (skin pigmentation, ageing) or behaviour (sunscreen use, cultural reasons). However, there are a few natural foods rich in vitamin D. Thus, vitamin D-enriched foods produced through a food chain approach such as feeding animals vitamin D supplements or vitamin D-fortified foods are needed to guarantee an adequate dietary intake of vitamin D by the general population.

The present review summarised the available and limited number of RCT investigating the effect of 25(OH)D_3_ supplementation on serum or plasma 25(OH)D concentration. We concluded that it is difficult to get consensus on the effectiveness of 25(OH)D_3_ supplementation relative to vitamin D_3_ for raising vitamin D status, due to various influencing factors such as different person characteristics (age, BMI), baseline vitamin D status and time of the year. However, it is unquestionable that 25(OH)D_3_ supplementation is more efficient at raising serum 25(OH)D concentrations and also appears to be absorbed faster by than the same dose of vitamin D_3_. Second, by reviewing available evidence on vitamin D-enriched eggs, fish or milk, it is practical and possible to increase the vitamin D content of eggs, fish or milk by addition of vitamin D supplements to the diet of poultry, fish or dairy cows. However, the limitations of adding vitamin D to animal feed should be considered in future enrichment studies. Furthermore, there are a few RCT investigating the impact of these vitamin D-enriched foods on improving vitamin D status. Therefore, 25(OH)D_3_-enriched or fortified foods should be further explored in the future, and additional RCT should be conducted to investigate the effect of 25(OH)D_3_-enriched or fortified foods on vitamin D status of the general population and patients with long-term health conditions.
